# COX2-ATP Synthase Regulates Spine Follicle Size in Hedgehogs

**DOI:** 10.7150/ijbs.83387

**Published:** 2023-09-04

**Authors:** Man Zhang, Mengyue Wang, Jingwei Jiang, Weiwei Liu, Siyi Zhou, Dehuan Wang, Miaomiao Wang, Zixian Zhao, Zhiling Xu, Wang Wu, Xia Lin, Jinwei Zhang, Wei Xu, Qu Tang, Rixing Zhan, Wanqian Liu, Li Yang, Xun Zhou, Wei Zhou, Mingxing Lei

**Affiliations:** 1 111 Project Laboratory of Biomechanics and Tissue Repair & Key Laboratory of Biorheological Science and Technology of Ministry of Education, College of Bioengineering, Chongqing University, Chongqing 400044, China.; 2 Three Gorges Hospital, Chongqing University, Chongqing 404000, China.; 3 Department of Dermatology and Cosmetology, The First Affiliated Hospital of Chongqing College of Traditional Chinese Medicine, Chongqing 400021, China.; 4 Institute of Burn Research, State Key Laboratory of Trauma, Burn and Combined Injury, Southwest Hospital, The Third Military Medical University, Chongqing 400038, China.; 5 Chongqing Key Laboratory of Translational Research for Cancer Metastasis and Individualized Treatment, Chongqing University Cancer Hospital, Chongqing 400030, China.

**Keywords:** ATP synthase, COX2, Hair follicle, Size control, Cell proliferation

## Abstract

Skin evolves essential appendages with adaptive patterns that synergistically insulate the body from environmental insults. How similar appendages in different animals generate diversely-sized appendages remain elusive. Here we used hedgehog spine follicles and mouse hair follicles as models to investigate how similar follicles form in different sizes postnatally. Histology and immunostaining show that the spine follicles have a significantly greater size than the hair follicles. By RNA-sequencing analysis, we found that ATP synthases are highly expressed in hedgehog skin compared to mouse skin. Inhibition of ATP synthase resulted in smaller spine follicle formation during regeneration. We also identified that the mitochondrial gene COX2 functions upstream of ATP synthase that influences energy metabolism and cell proliferation to control the size of the spine follicles. Our study identified molecules that function differently in forming diversely-sized skin appendages across different animals, allowing them to adapt to the living environment and benefit from self-protection.

## Introduction

In the history of evolution, animals have evolved with unique characteristics to adapt to the ever-changing environment. Different adaptive patterns in animals may play different yet important roles in survival, reproduction, defense, food-hunting, etc. Locating on the surface of the body, skin generates obvious and diverse patterned appendages including follicles (e.g. hair, feather, and spine), glands (e.g. sebaceous, sweat, and mammary), and keratinized structures (e.g. scale, claw, and horn) that function importantly for animals, such as barrier and protection, thermoregulation, communication, hunting, defense, lighting, and secretion 1. The hair follicle is a highly conserved mini-organ that mainly functions on thermoregulation and also roles in the immune response against pathogens 2. Most mammals are covered with hair follicles over 90% of the body surface 3. Interestingly, the diverse animal kingdom evolves with essential skin appendages that endow animals with specialized functions. For example, different from mice which are cave animals covered with hairs to keep warm, the hedgehog is a heterothermic animal with dorsal skin covered with hard and sharp spines that are powerful for defense when subjected to environmental insult 4, 5. While the hair follicle and spine follicle have similar morphology, the mechanism by which the different size of these follicles remains unknown.

The control of organ size is fundamental in living life 6. The size of an organ directly determines the function of the organ. The hair follicle is a mini-organ that changes its size during cycling including the growth phase (anagen), regression phase (catagen), and resting phase (telogen). The hair is produced by the hair follicle. The hair length is determined by the duration of the growth phase and the hair diameter is controlled by the size of the hair bulb and dermal papilla (DP) 7, 8. In mouse hair follicles, VEGF improves hair follicle vascularization to promote hair growth and increase hair follicle size 9. Wnt10b regulates the size of the hair bulb, dermal papilla, and hair shaft during hair regeneration in mice, and leads to the change of CD34 expression 10. BMP controls hair follicle size by regulating cell cycle-related genes and changing cell proliferation 11. In particular, it was also found that the number of DP cells also played a decisive role in controlling hair size and shape, and DP secretes signals to activate stem cells to start the formation of new hair follicles 12. Activators secreted by white adipose tissue (dWAT) near hair follicles can also change the state of hair follicle stem cells, thus regulating the speed of hair regeneration 13. Similar to the hair follicle, the hedgehog spine follicle is also developed during embryogenesis that involves epithelial-mesenchymal interaction, but the latter one grows into bigger follicles during development and forms big spines postnatally. While the molecular mechanism involved in regulating hair follicle size has been gradually uncovered, it is interesting to elucidate the mechanism by which the similar spine follicle in the hedgehog grows into a significantly bigger follicle in size, which allows the hedgehog to accommodate the environment.

Mitochondrially encoded cytochrome c oxidase II (COX2) is a subunit of cytochrome c oxidase (complex IV), an enzyme that drives oxidative phosphorylation in the mitochondrial electron transport chain. Cytochrome c oxidase is at the end of the cytochrome system in cellular respiration. It transfers electrons from NADH and succinic acid to oxygen, and produces an electrochemical gradient on the intima. The electrochemical gradient drives transmembrane transport and ATP synthesis. Energy metabolism in mitochondria is crucial for hair follicle development 14. The study found the activity of electronic transport chain complexes (complexes I, IV, and V) and the ability to produce adenosine triphosphate (ATP) are significantly decreased in the dermal papilla of male hormone alopecia, indicating mitochondrial function is impaired in dermal papilla 15. In addition, the inactivation of cytochrome c oxidase is also the main metabolic defect of the dermal papilla, which will lead to the reduction of ATP production, the depolarization of mitochondrial membrane potential, the increase of reactive oxygen species (ROS) level, and the reduction of the key marker expression in dermal papilla 16. The disorder of cytochrome c oxidase will lead to a change in ATP synthesis and affect physiological phenomena. ATP synthase is a nanometric rotary machine mainly located on the inner membrane of mitochondria, playing roles in development, differentiation, and cell proliferation 17, 18. ATP synthase can form ATP by transmembrane electrochemical gradient and powers the cell 19, 20. The expression of ATP synthase is related to the differentiation of keratinocytes. ATP synthase subunit ATP5B is increased during keratinocyte differentiation in normal skin and some epidermis hyper-proliferative diseases 17.

In this study, we study how hedgehog forms bigger spine follicles in size with mouse hair follicles as a comparison. We first characterized the epithelial and mesenchymal components in the hedgehog spine follicle. By RNA-seq analysis of growth phase follicles in hedgehogs and mice, we identified genes including ATP synthase that are specifically expressed in the hedgehog spine follicles. Functional perturbation of ATP synthase resulted in smaller spine follicle formation. Then we investigated the upstream regulator of ATP synthase with a focus on a mitochondrial gene COX2, inhibition of which also led to a dramatically decreased size of spine follicle at the full growth phase. Through comparison between spine follicles in hedgehogs and hair follicles in mice, our study gains new insight into understanding the adaptative mechanism by which the diversity in skin appendages forms between different species in nature.

## Materials and Methods

### Animals

C75BL/6J mice were used in this study. Adult mice were used to study hair follicles and 45-day hedgehogs (Atelerix Albiventris) purchased from Jianghu Pet Corporation (Yiyang, China) were used to study spine follicles.

### Phenotypic analysis

To observe the differences between hairs in mice and spines in hedgehogs, their back skins were directly dissected and photographed with a camera (Canon) or stereoscopic microscope.

The spines and hairs were plucked out from the skin to measure the length and width. The microstructure of the spine and hair was observed with a microscope and photographed with NScope 2.0. The length and width of the spine follicles and hair follicles at full growth phase were photographed with a stereoscopic microscope, and measured by Adobe Photoshop CC 2019.

### BrdU administration

For pulse labeling, BrdU (Beyotime, Sigma) was administered by injecting intraperitoneally at 50 mg per kg (body weight). The injection was done 4 hours before collecting spine and hair follicles samples.

### Histology, hematoxylin, and eosin (H&E) staining, and immunohistochemistry

Dorsal skin samples were fixed for 48 hours in 4% paraformaldehyde (PFA) at 4°C, washed with PBS, and dehydrated with gradient alcohol (50%, 75%, 85%, 95%, and 100%) and xylene. After mounting in paraffin, samples were sectioned at 8 µm in thickness.

For H&E staining, samples were stained with hematoxylin for 3 minutes and eosin for 1 minute at room temperature, then dehydrated and mounted with resinene.

For immunohistochemistry, the slides were immersed in 10 mM sodium citrate buffer (pH 6.0) at 100°C. Then slides were blocked with 1% bovine serum albumin (BSA) buffer at 37oC for 1h, incubated with primary antibodies (K14, K17, Vimentin, Collagen I, PCNA, and BrdU) at 4°C overnight, followed by incubation with secondary antibodies (Alexa 647- and Alexa 488-conjugated anti-rabbit, anti-mouse, and anti-goat, Beyotime, ZSGB-BIO) at 37^o^C for 2 hours. Primary antibodies were diluted at 1:100 and secondary antibodies were diluted at 1:500 or 1:50 in PBS. The samples were counterstained with propidium iodide (PI) or 4'6'-diamidino-2- phenylindole (DAPI) to label the nuclei.

### Small molecules perturbation

ATPase inhibitor (Blebbistatin, 1mM, Beyotime) and COX2 inhibitor (NS-398, 50 µM, Beyotime) were injected into the bottom of the spine follicle at the early growth phase using a microinjector, with DMSO as a control. Spine follicles were injected for two consecutive days and collected on the third and ninth days to observe their phenotypes using HE and immunofluorescence staining.

### Transcriptome analyses

Two available hedgehog skin transcriptome data were downloaded from Hui-Ming Li, 2020 4, SRA experiment accessions are SRX6788248 and SRX6788249 respectively. In this study, the whole dorsal back skin with the spine follicles at the growth phase in hedgehogs was collected for transcriptome analysis. Two available mice skin transcriptome data were downloaded from previous studies 21. In this study, the whole dorsal back skin with the hair follicles at the growth phase in mice was collected for transcriptome analysis. Then the basic local alignment search tool (BLASTX) was used to annotate all unigenes. By analyzing the intersection situation between the genes in hedgehogs and mice, the genes specifically expressed in hedgehogs were screened out.

The genes only expressed in hedgehog were analyzed and expression value > 1 and standard deviation < 100 were set as the threshold. Expression value means read count. The higher the read count, the higher RNA in abundance. The standard deviation is used to judge the statistical dispersion of gene expression levels in the replicates of RNA-seq data. STRING 22 (Version: 11.5) was run for gene ontology (GO) and Kyoto Encyclopedia of Genes and Genomes (KEGG) enrichment analysis. The relationship of genes in the pathways was also analyzed by STRING.

We used statistical software R (version 4.0.3) for visualization. R-package “ggplot2” and “ggbreak” were used to analyze and show Boxplot and Volcano map. Heatmap, GO and KEGG enrichment results were plotted by bioinformatics 23, an online platform for data analysis and visualization. Venn diagrams were prepared using an online website (Bioinformatics & Evolutionary Genomics) 24. The sector diagram is drawn in Excel.

### Statistical analyses

The counting tool in Photoshop is used to label each proliferating cell and the number of proliferating cells is obtained.

All experiments were repeated at least six times. Statistical significance was evaluated using the Student's t-test. P values of less than 0.05 were used as the threshold to determine the significance.

## Results

### Characteristics of spine follicles in hedgehogs and hair follicles in mice

**Figure 1:**
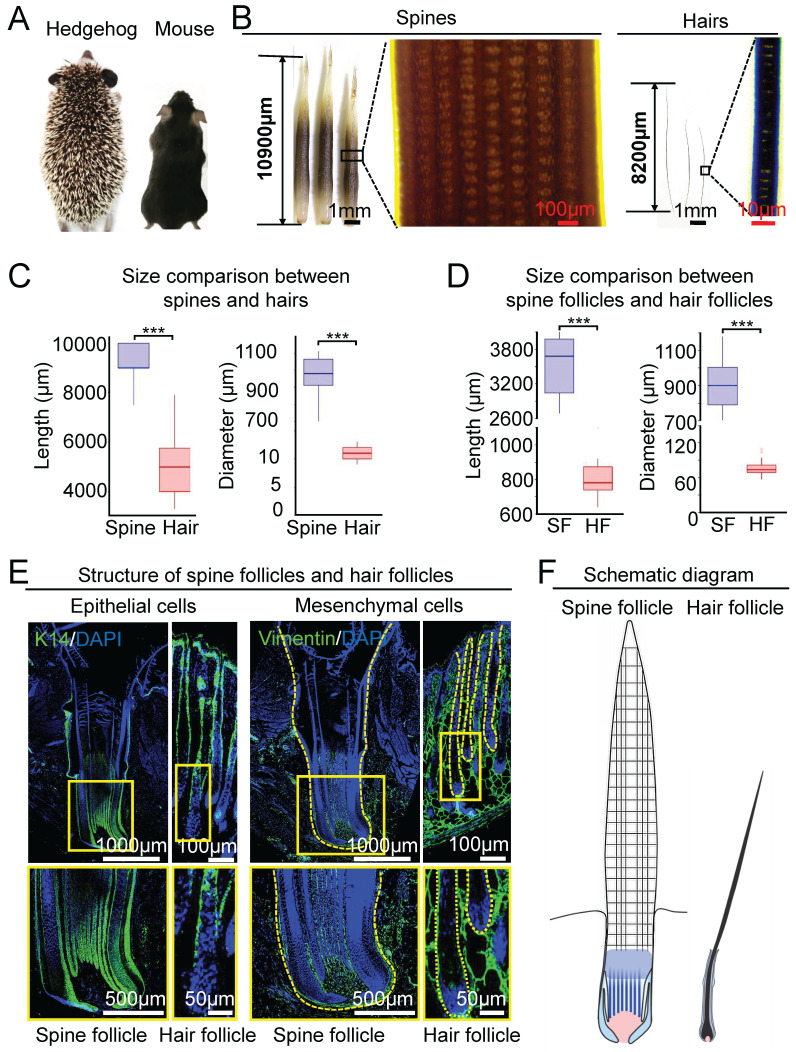
Comparison of the size between the spine follicles in hedgehog and the hair follicles in mouse. A. Dorsal skin appendages in hedgehog and mouse. B. Size difference in spines and hairs at full-growth phase. C. Statistical chart shows the length and diameter of spines in hedgehogs and hairs in mice. D. Statistical chart shows the length and diameter of spine follicles in hedgehogs and hair follicles in mice. E. Immunostaining for K14 and Vimentin shows the epithelial and mesenchymal cells in the spine follicle and hair follicle. F. Schematic of the spine follicle and hair follicle. SF: spine follicle; HF: hair follicle.

To show the differences between hedgehog spine and mouse hair on the dorsal back skin, we compared the length and diameter of the spine and hair at the full-growth phase, when they reach their biggest size (Figure 1A-B, Figure S1A). The hedgehog spine is longer and thicker than the mouse hair (Figure 1B, Figure S1B). Phase-contrast microscopy and statistical analysis show that the length of the spine (9.26±0.68 mm) is significantly longer than the mouse hair (5.03 ± 1.34 mm) (Figure 1B-C), and the average diameter of the spine (0.96 ± 0.12 mm) is also significantly thicker than the hair (10.89±1.17 µm, Figure 1B-C).

Spines and hairs are produced hair their follicles embedded in the skin. We then compared their follicle morphology and size at the full-growth phase. HE staining showed that the spine follicle and hair follicle have similar structures mainly including the epithelial and mesenchymal components, which were verified by immunostaining for K14 and Vimentin, respectively (Figure 1E). There are two major differences between hedgehog spine follicles and mouse hair follicles. One is the size of the follicle which can be judged by the length and diameter. The average length is 3.50 ± 0.50mm in the spine follicle and 0.80 ± 0.1 mm in the mouse hair follicles (Figure 1D). The average diameter is 0.91 ± 0.14 mm in the spine follicle and 77.68 ± 13.79 µm in mouse hair follicles (Figure 1D). Thus, the diameter of the spine follicle is 11 times greater than that of hair follicles. The second major difference is that the epithelial component of the spine follicle includes the ridges that intermingled with dermal pulp cells, whereas the hair follicle only contains that hair medulla in a relative location (Figure 1E, Figure S1B-C). Together, these data indicate that the hedgehog spine follicle is significantly larger than the hair follicle, which generates a bigger spine and hair in size, respectively (Figure 1F).

**Figure 2:**
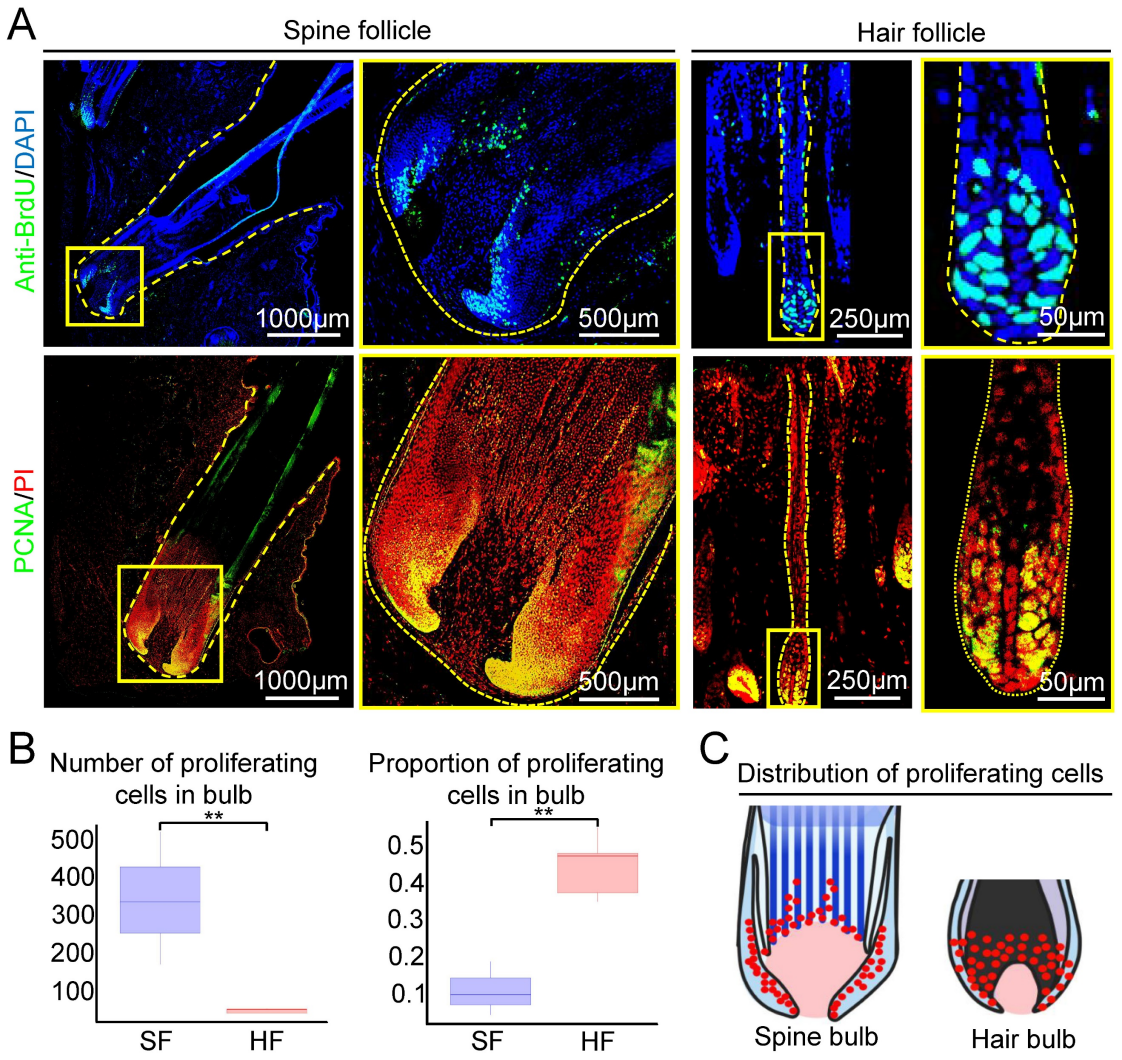
The number and distribution of proliferating cells in spine follicles and hair follicles at the growth phase. A. Proliferating cells in spine follicles and hair follicles. Immunostaining for BrdU shows label-retaining cells (LRCs). Immunostaining for PCNA shows proliferating cells. B. Number and proportion of proliferating cells in the bulb (**p < 0.01). C. Schematic diagram shows the proliferating cells in the spine follicle and hair follicle.

To explore if the formation of large spine follicle formation is due to increased cell proliferation, we examined the number and distribution of proliferating cells in spine follicles and hair follicles at the growth phase using BrdU labeling and PCNA immunofluorescence staining. The majority of proliferating cells are distributed in the bulb region in both hair and spine follicles (Figure 2A). Spine follicle has nearly 10 times more proliferating cells (332 ± 48.46) than that of the hair follicle (33.6 ± 5.4, Figure 2A-B). Interestingly, only 11% of cells are proliferating in the bulb region of the spine follicle but 45% in the hair bulb (Figure 2B). We also make statistics on proliferating cells in the dermal papillae (DP). The average number of proliferating cells in the spine follicle DP is 25 ± 7.36, but 1 ± 1.41 in the hair follicle DP (Figure S2). These data indicate that the number of proliferating cells might be one of the factors that affect the size of the spine follicles (Figure 2C).

### Transcriptome profiling of spine follicles in hedgehogs and hair follicles in mice

**Figure 3:**
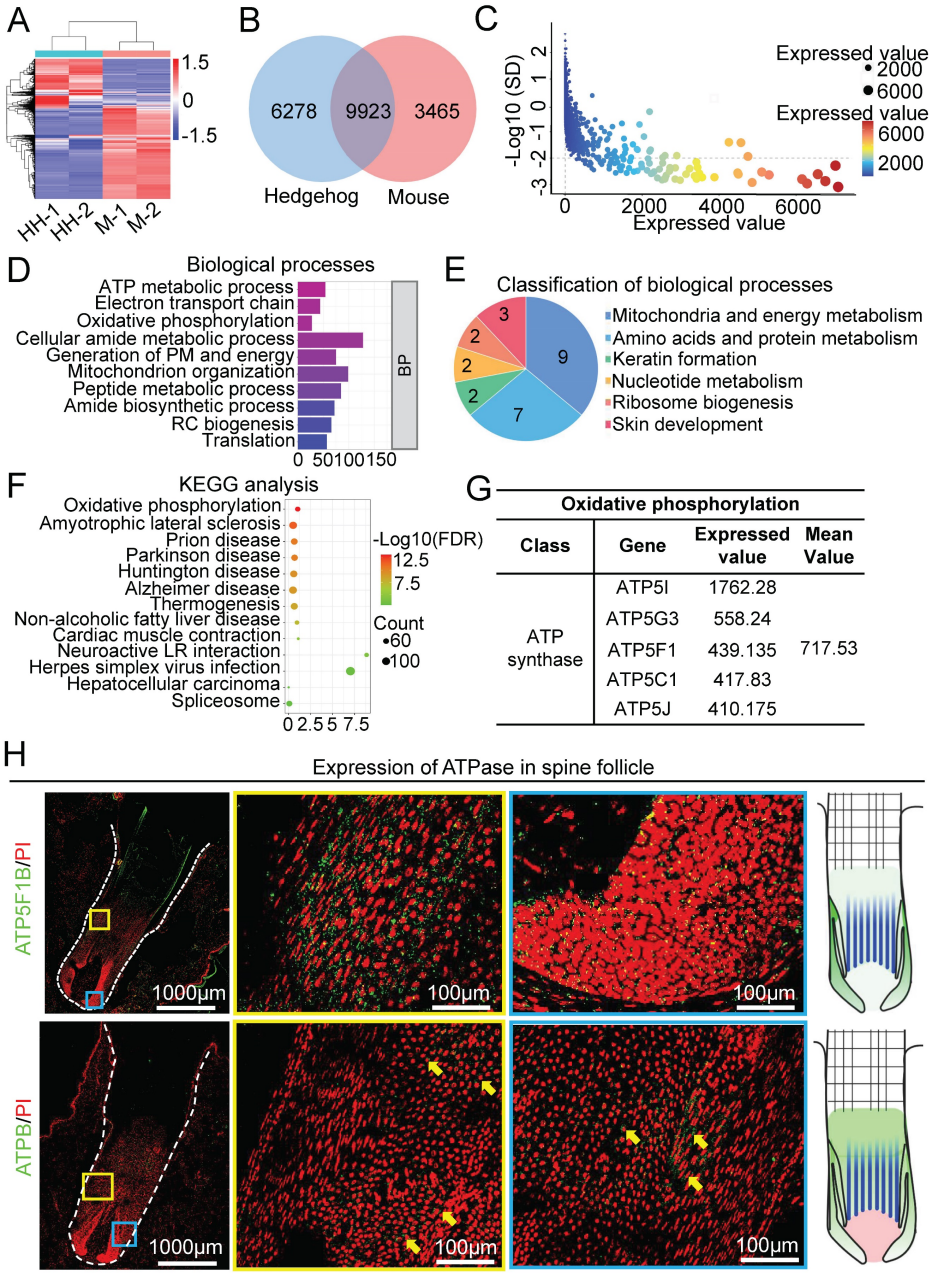
RNA-seq profiling and bioinformatics analysis reveal key molecules at the growth phase of the spine follicles. A. Heatmap shows gene expression of spine follicles in hedgehogs and hair follicles in mice at the growth phase (HH: hedgehog, M: mouse). B. The overlap of genes in hedgehogs and genes in mice. C. The genes were only expressed in the spine follicles. SD: standard deviation. D. Biological processes of genes only expressed in the spine follicles. E. Pie chart shows the classification of biological processes. F. KEGG pathway analysis of genes only expressed in the spine follicles. G. The expression value of genes encoding ATP synthase. H. The expression position of ATPase in the spine follicles. Green shows the expression of ATP5F1B.

To identify the molecular difference between spine follicles and hair follicles that may contribute to their different sizes, we analyzed the transcriptome by RNA-sequencing of growth phase follicles (Figure 3A). Venn diagram shows that 9923 genes were expressed in both mouse and hedgehog follicles, whereas 6278 genes were only expressed in the spine follicles and 3465 genes were only expressed in the hair follicles (Figure 3B). Since previous studies have elucidated the molecular mechanism of hair follicle size control 7, 10, we tried to identify the molecules which may regulate spine follicle size. By selecting genes with an expression value greater than 1 and the standard deviation between the two samples less than 100 as the threshold, we distilled 4103 genes for further analysis (Figure 3C).

Biological processes of gene ontology (GO) revealed that 9 terms were related to mitochondria and energy metabolism (Figure 3D-E, Figure S3A). These terms were also included when performing cell composition and molecular function GO analysis (Figure S3B). KEGG pathway analysis of these genes revealed that oxidative phosphorylation ranks first among all terms (Figure 3F), with 16 related genes in this pathway (Figure S3C-D). The proteins encoded by these 16 genes are the subunits of ATP synthase, Inorganic pyrophosphatase, ubiquinone oxidoreductase, and V-ATPase (Figure S3D). The genes encoding ATP synthase include ATP5I, ATP5G3, ATP5F1, ATP5C1, and ATP5J, with a relatively higher expression among the other three gene groups (Figure 3G, Figure S3E). To verify the expression of ATPase in the spine follicle, we stained ATP5F1B and ATPB in the spine follicle. ATP5F1B is a gene encoding ATPase in the spine follicle and ATPB is a subunit of ATPase that is expressed in the outer root sheath of the spine follicle (Figure 3H). Oxidative phosphorylation is important for energy metabolism in mitochondria, and the high expression of genes related to ATP synthase may suggest that ATP synthase plays an important role in modulating hedgehog spine follicle formation.

### ATP synthase influences spine follicle size in the growth phase

**Figure 4:**
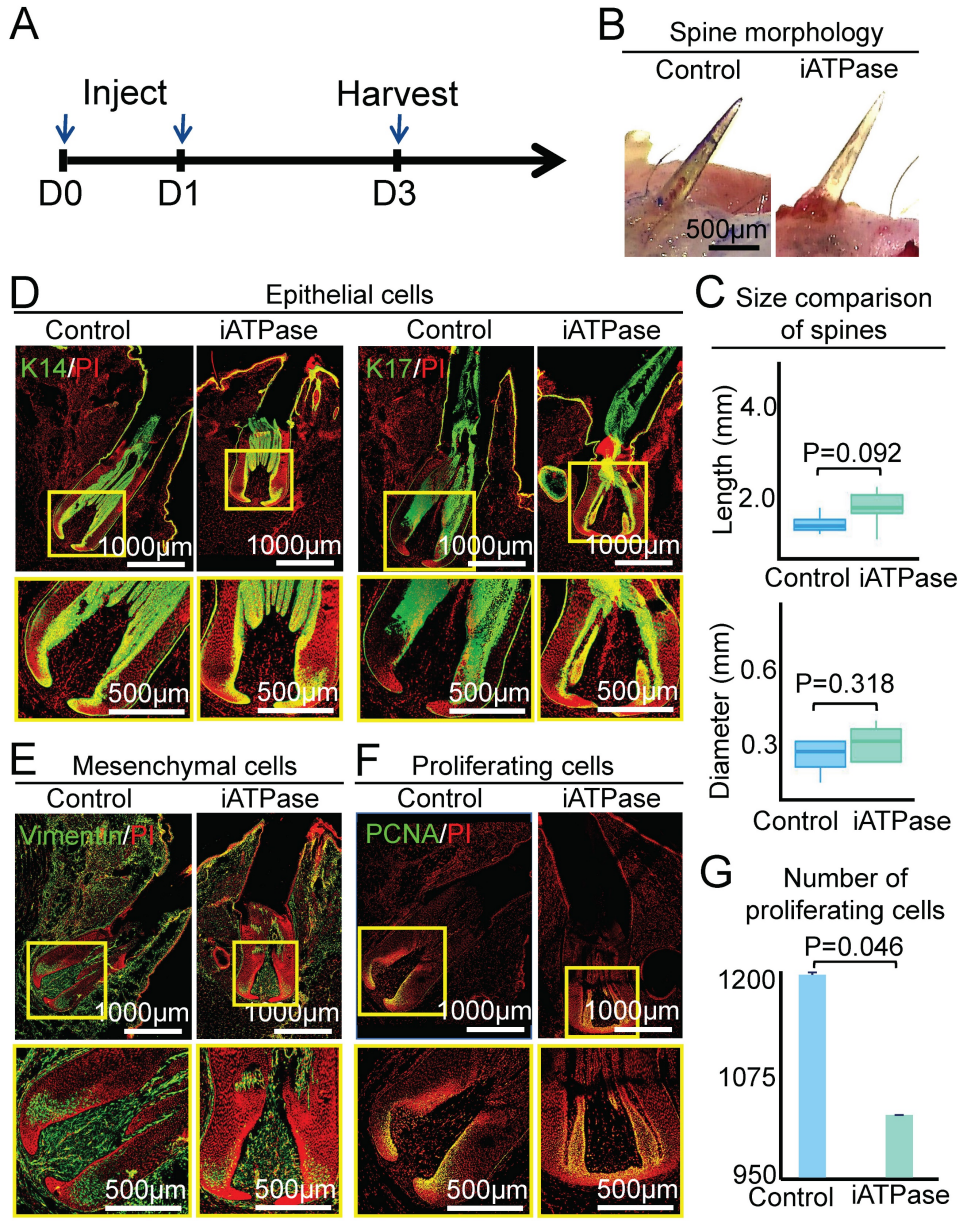
ATP synthase does not change spine follicle size on the third day after injection of Blebbistatin. A. Experimental design. B. Spine morphology outside of the skin. C. statistical chart shows the length and diameter of the spines in the control group and the experimental group. D. Immunostaining for K14 and K17 shows the internal structure of epithelial cells. E. Immunostaining for vimentin shows the internal structure of mesenchymal cells. F. Immunostaining presenting changes in the number of proliferating cells. G. Statistical chart presenting the number of proliferating cells.

**Figure 5:**
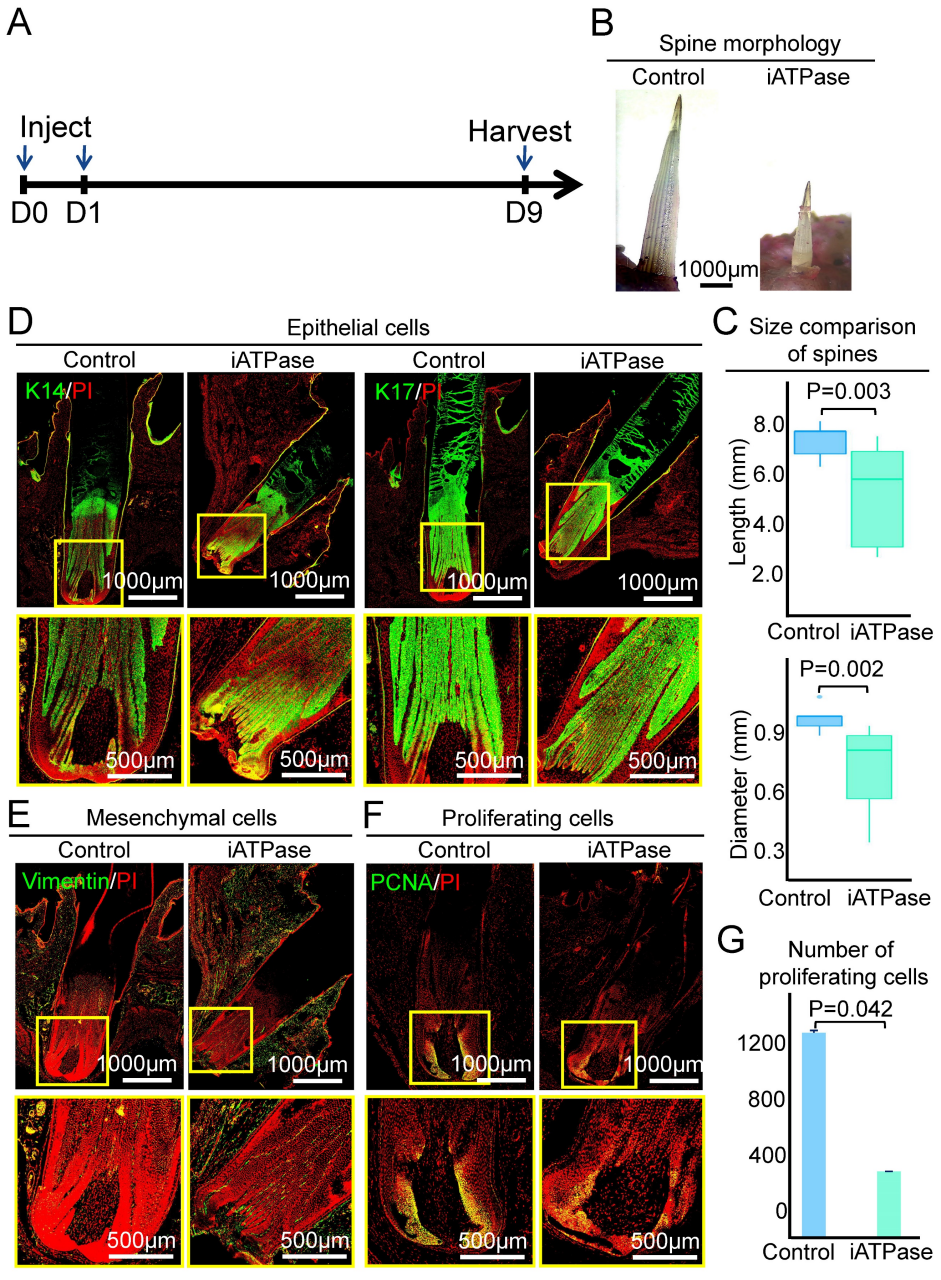
Inhibiting ATP synthase reduces spine follicle size on the ninth day after injection of Blebbistatin. A. Experimental design. B. Spines outside of the skin are shorter and thinner. C. statistical chart shows the length and diameter of the spines in the control group and the experimental group. D. Immunostaining for epithelial cells shows that the spine follicles in the experimental group were smaller. E. Immunostaining for mesenchymal cells shows that the spine follicles in the experimental group were smaller. F. Immunostaining presenting proliferation cells decrease in the experimental group. G. Statistical chart presenting the number of proliferating cells.

To determine if ATPase affects the size of the spine follicle, we intracutaneously injected Blebbistatin (an ATP synthase inhibitor) 25 at the initial growth phase of the spine follicle and observed the morphology of the spine and spine follicle at D3 and D9, which represent early growth phase and full growth phase, respectively (Figure 4A, Figure 5A). First, we detected the expression level of ATPase-related genes in the spine follicle by qPCR to determine if Blebbistatin inhibited ATPase. The result shows that the expression of ATP5G3, ATP5F1, and ATP5J in the Blebbistatin-treated group (iATPase) was significantly lower than those in the control group (Figure S4A). Second, we further validated the distribution of ATPase subunit ATPB by immunofluorescence staining (Figure S4B). Immunofluorescence staining shows that the expression of ATPB was significantly lower in the Blebbistatin-treated group (iATPase) than those in the control group. These results suggest that Blebbistatin can inhibit ATPase in the spine follicle. Then we observed the changes in the size of the spine follicle after the inhibition of ATPase. On the third day after injection of Blebbistatin, we observed there was no obvious change in the spine outside of the skin (Figure 4B-C, P = 0.092), with an average length of 1.10 ± 0.19 mm in the control group and 1.41 ± 0.31 mm in the Blebbistatin-treated group. The average diameter of the follicles in the two groups was also not dramatically changed (Figure 4C, P = 0.318). HE staining and immunofluorescence staining showed that the follicle structure was not changed (Figure 4D-E, Figure S4C-D).

However, the spine in the experimental group was significantly shorter and thinner than the control group when the follicle entered the full growth phase at D9. The average length and diameter of the spine have reached 7.52 ± 0.66 mm and 0.99 ± 0.07 mm (P = 0.003), respectively, while the average length and diameter of the experimental group were 5.32 ± 1.86 mm and 0.73 ± 0.21 mm (P = 0.002), respectively (Figure 5B-C). The spine follicle length was 2.84±0.30mm in the experimental group and 3.65 ± 0.12 mm in the control group, and the diameter was less than that in the control group (0.93 ± 0.03 mm) (Figure 5D-E, Figure S5A-B). These data indicate that ATP synthase plays an important role in regulating the size of the spine follicle which further influences the spine size.

We then used PCNA immunostaining to determine if ATP synthase influences spine follicle size by regulating cell proliferation. Interestingly, while the spine follicle didn't change in size at D3, the number of PCNA+ cells in the blebbistatin-treated follicle (1040 ± 17) is significantly decreased than that in the control group (1175 ± 25), particularly in the bulb region (Figure 4F-G). The number of proliferating cells is also dramatically decreased in the blebbistatin-treated follicle (716 ± 48) compared to the control group (1212 ± 12) at D9 (Figure 5F-G). This suggests that ATP synthase indeed functions in regulating cell proliferation in spine follicles.

### The function of the mitochondrial gene - COX2

**Figure 6:**
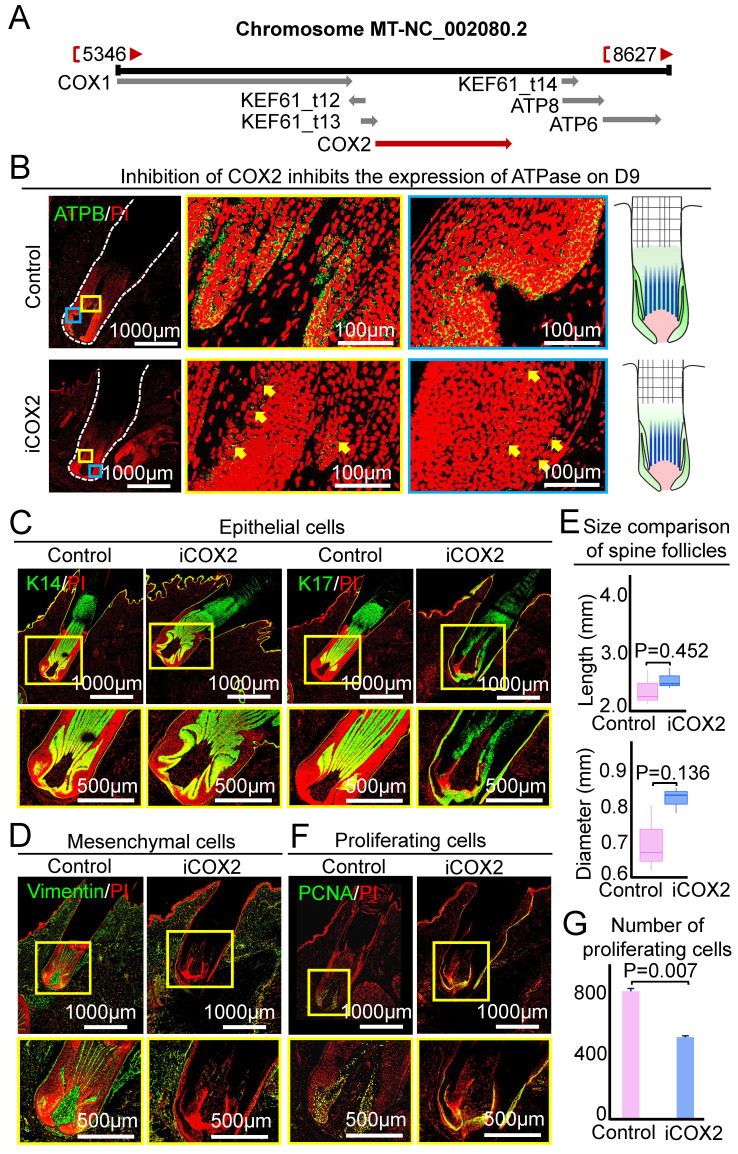
COX2 does not change spine follicle size on the third day after NS-398 treatment. A. Localization of COX2 in hedgehog genome. B. Changes of ATPase expression on the ninth day. Green shows the expression of ATPB. C. Immunostaining for K14 and K17 shows the internal structure of epithelial cells. D. Immunostaining for vimentin shows the internal structure of mesenchymal cells. E. Statistical chart shows the length and diameter of spine follicle on the third day. F. Immunostaining presenting changes in proliferating cells. G. Statistical chart presenting the number of proliferating cells.

The role of ATP synthase is to synthesize ATP and generate energy in mitochondria. And ATP synthase upstream pathway is the respiratory chain in mitochondria, which transfers electrons to generate oxygen into the water. Therefore, the respiratory chain plays an important role in regulating ATP synthase. The last enzyme in the respiratory chain is cytochrome c oxidase. And previous study shows that COX2 can regulate ATP synthesis in rat liver mitochondria 26. COX2 is a mitochondrial gene in hedgehogs (Figure 6A), we used COX2 small molecule inhibitor (NS-398) to test if this gene influences the growth of the spine follicle. The expression of ATPB in the experimental group was significantly decreased than that in the control group on the ninth day, indicating that COX2 can regulate the expression of ATP synthase in the spine follicle (Figure 6B). Similar to the inhibition of ATP synthase, injection of NS-398 resulted in no significant change in the size of the spine and spine follicle compared to the control at D3 (Figure 6C-E, Figure S6A-B).

**Figure 7:**
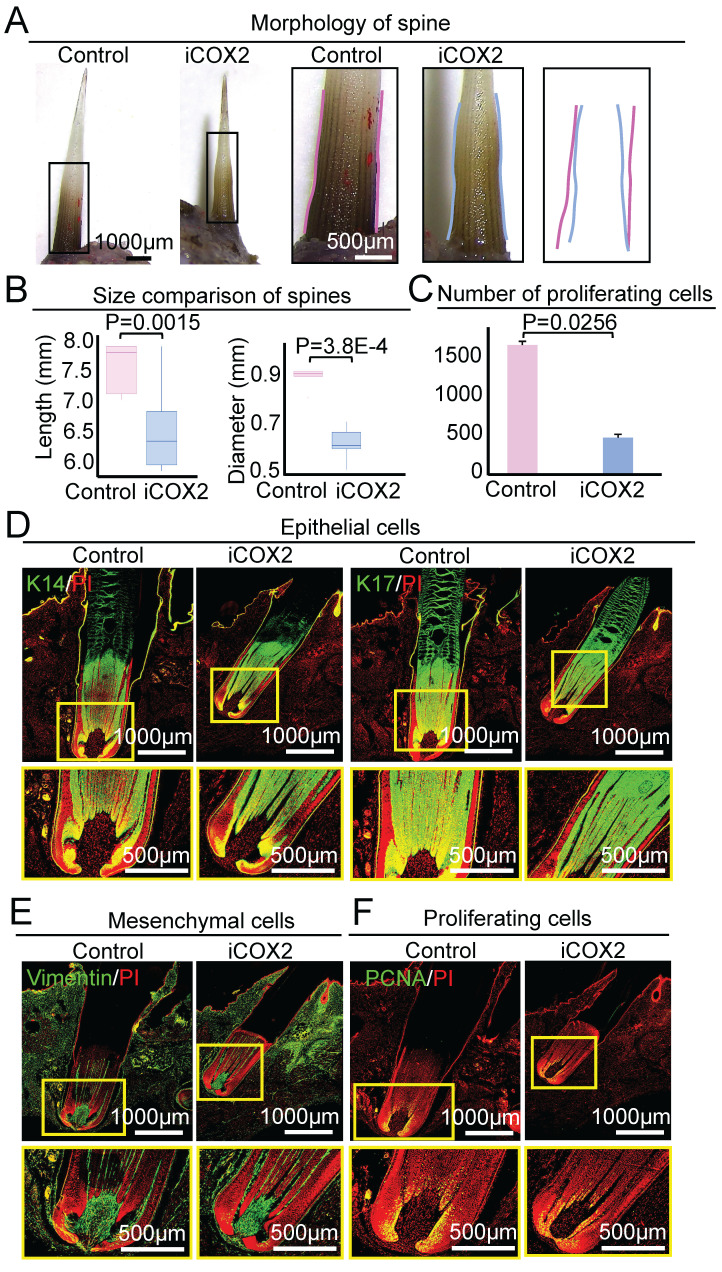
Inhibiting COX2 reduces spine follicle size on the ninth day after NS-398 treatment. A. Spines outside of the skin are smaller. B. statistical chart shows the length and diameter of the spines in the control group and the experimental group. C. Statistical chart shows proliferation cells decrease in the experimental group. D. Immunostaining for epithelial cells shows that the spine follicles in the experimental group were smaller. E. Immunostaining for mesenchymal cells shows that the spine follicles in the experimental group were smaller. F. Immunostaining for PCNA presenting proliferation cells in spine follicle.

In contrast to the control group, the spine of the experimental group became significantly smaller in size after NS-398 treatment on D9 (Figure 7A). The average length of the spine was 7.54 ± 0.40mm and 6.41 ± 0.58 mm in the control group and the experimental group, respectively (P = 0.0015). The average diameter of the spine was 0.91 ± 0.08mm and 0.61 ± 0.07 mm in the control group and the experimental group, respectively (P = 3.8E-4, Figure 7B). HE staining and immunofluorescence staining showed that the spine follicle in the experimental group (Length = 3.58 ± 0.31 mm, Diameter = 0.86 ± 0.02 mm) was significantly smaller in size than the control group (Length = 4.44 ± 0.23 mm, Diameter = 0.99 ± 0.05 mm) (Figure 7D-E, Figure S7A-B). These data suggest that COX2 functions by regulating ATP synthase in mitochondria to modulate spine follicle size.

We also used PCNA immunostaining to examine if COX2 influences spine follicle size by regulating cell proliferation. The number of PCNA+ cells in the NS-398-treated follicle is significantly decreased than that in the control group, particularly in the bulb region at D3 and D9 (Figure 6F-G, Figure 7C-F). This suggests that COX2 also functions in regulating cell proliferation in the spine follicle.

## Discussion

Diverse skin appendages such as scales, feathers, and hairs are generated in amniotes to form the boundary between the animal and the environment. Interestingly, novel functions evolve in structurally-similar appendages in different animals which enable organisms to adapt to terrestrial conditions. The hair follicle in mice and spine follicles in hedgehogs have similar follicular morphology but with different sizes that allow them to adapt to the environment differently, for example, keeping warm for mice and defensive response for hedgehogs. In this study, we studied the mechanism by which the hedgehogs form bigger spine follicles with mouse hair follicles as a comparison. We unveiled that the COX2-ATP synthase axis controls the size of spine follicles by regulating cell proliferation in hedgehogs.

### Cell proliferation regulates organ size in living organisms

The control of organ size is a central question in biology 6. Despite the attention it has been paid, the mechanism that how adult organ size is determined is still incomplete. Cell proliferation is involved in the regulation of organ size 27, 28. Cells extensively divide when an organism is growing. Whereas in adulthood, although cells continue dividing, the size of organs remains fixed. This is because the number of apoptotic cells and dividing stem cells is always consistent 29. For example, E-cad—Rho—Egfr has been elucidated in the adult Drosophila that controls a constant intestine size 30. Apoptotic enterocytes conversely promote cell division by loss of E-cadherin, which leads to an increased expression of rhomboid and triggers activation of the EGF receptor in stem cells which then proliferate. On the contrary, E-cad—Rho—Egfr inhibits stem cell division when apoptosis ceases. Thus, organisms can maintain a constant organ size through this precise regulation by balancing cell proliferation and apoptosis.

The present study showed that, although hair follicles in mice and spine follicles in hedgehogs are similar skin appendages in morphology, their sizes can be prominently different. At the full growth phase, the spine follicle is 4 times longer than the hair follicle in length and the diameter is more than 10 times in width. We show that it is because of a significant increase in cell proliferation number but not proliferation rate that leads to enlarged spine follicles in hedgehogs, compared to the hair follicles in mice. Our study identified how cell proliferation controls differently-sized organ formation in different animals. Indeed, this is common in living animals. For example, with the decline of cell proliferation, the liver volume of mice and rats tends to be stable and maintains a specific size. The proliferation in the liver of mice and rats leads to a huge difference in the number of cells, which leads to a difference in liver size 31.

Although the size of organs in an individual is constant in adulthood, the size of organs in different individuals of the same species often varies, and this is affected by many factors such as age, sex, height, and body weight. In humans, axial eye length is positively correlated with height and weight 32, while eye weight is positively correlated with body weight in mice 33. The chicken eye size is also related to body size, and about half of the eye size variation is determined by pleiotropic genes that also affect body size 34. Since body weight is significantly higher in hedgehogs than in mice, it is also interesting to study if the determinant of spine follicle size has to be correlated to body weight.

Hair follicles in humans, mice, goats, and birds have similar morphological structures and share similarities in the growth cycle. With the interaction between mesenchymal and epithelial cells, they all undergo cyclic regeneration regulated by various factors. Current research indicates that the signals that regulate growth and regeneration are similar, including Wnt, SHH, and BMP, but the regulatory mechanisms of these signals vary among different species and can lead to the formation of different morphological architectures. The hedgehog spine follicle is also one of the skin appendages, and the morphological regulation during the regeneration process of the spine follicle may also be controlled by the same signaling pathways. Thus, it is intriguing to study the specific controlling mechanism in hedgehogs or other animals with skin appendages in future studies.

### COX2-ATP synthesis regulates cell proliferation that modulates spine follicle size

While the size of hair follicles has been studied, we investigated the mechanism by which size control is regulated in spine follicles in the present study. The bioinformatic analysis and functional experiments in our study indicate that ATP synthase and COX2 have a more specific expression in spine follicles compared to the hair follicles, and inhibition of them resulted in a smaller spine follicle and spine in size. However, inhibitors cannot accurately target the location of the spine follicle and may cause off-target effects. While the transgenic technology for hedgehogs is difficult to achieve, we may use lentivirus with specific promoters to activate or silence gene expression in future studies. ATP synthase is the key enzyme for energy metabolism that participates in oxidative phosphorylation 35, 36. Some ATP synthase-related genes are not expressed in mice but in hedgehogs. The high expression of these genes may change the subunit and quantity of ATP synthase and make the ATP synthase have different functions in hedgehogs. ATP synthase is involved in the synthesis of ATP which provides the energy needed for cell proliferation 18. Activation of Wnt/β-catenin signaling in DP cells increases the expression of proliferation marker PCNA, leading to an increased hair in length 37. Therefore, it is reasonable to speculate that increased expression of ATP synthase promotes the generation of ATP which provides the energy necessary for cell proliferation in spine follicles, leading to a larger spine follicle formation in hedgehogs.

COX2 inhibitors can inhibit ATP synthesis in rat liver mitochondria 26. When COX2 is up-regulated, it will cause hyperresponsiveness to ATP in guinea pigs 38. We also identified that COX2 functions as the upstream regulator of ATP synthase that regulates spine follicle size. Inhibition of COX2 led to the formation of smaller-sized spine follicles and spine in hedgehogs. COX2 is a mitochondrial gene in hedgehogs. COX2 is activated at the growth phase to increase the expression of ATP synthase-related genes in hedgehogs. This infers that increased ATP synthase in mitochondria may promote ATP synthesis, leading to increased cell proliferation and large spine follicle formation.

ATP synthase can provide energy for the sodium-potassium pump and power for K+ and Na+ to enter and exit cell, which is crucial for stem cell differentiation and organogenesis in multicellular organisms 39. Dermal papilla cells contain ATP-sensitive potassium (K(ATP)) channel in human hair follicles, and minoxidil may promote hair growth through the K(ATP) channel 40. In addition, research has found that hair follicle stem cells (HFSC) from aged eyelid skin can differentiate into corneal endothelial-like cells in humans, and these cells can maintain the electrochemical potential difference established by sodium/potassium ATPase, indicating that ATP synthase may play an irreplaceable role in this process 41. ATP synthase plays an important role in human hair follicles and controls hair follicle growth and hair follicle stem cell differentiation by providing energy for sodium and potassium channel. In hedgehog, after inhibiting the myosin II ATPase activity by Blebbistatin in the spine follicle, the size of the spine follicle was changed, indicating that ATP synthase may control the size of the spine follicle by providing energy for myosin.

## Conclusions

In conclusion, the present study reveals a novel mechanism by which the mitochondrial COX2 regulates ATP synthase to control spine follicle formation by regulating cell proliferation. Our study also suggests that, in addition to forming the adaptive pattern to accommodate the extrinsically ever-changing environment, the animals also evolve different intrinsic molecular regulatory systems that create biodiversity, including but not limited to the size control of the skin appendages.

## Supplementary Material

Supplementary figures.

## Abbreviations

COX2: Mitochondrially encoded cytochrome c oxidase II
DP: Dermal papillae
PFA: Paraformaldehyde
BSA: Bovine serum albumin
PI: Propidium iodide
DAPI: 4'6'-diamidino-2- phenylindole
LRCs: Label-retaining cells
HH: Hedgehog
M: Mouse
