# Supplementary material for: COX2-ATP Synthase Regulates Spine Follicle Size in Hedgehogs
Source: Int J Biol Sci. 2023 Sep 4;19(15):4763–77. doi: 10.7150/ijbs.83387 (PMC10539703; doi:10.7150/ijbs.83387)
Supplement: Supplementary file 1 — Supplementary figures. [file ijbsv19p4763s1.pdf]

Additional file 1

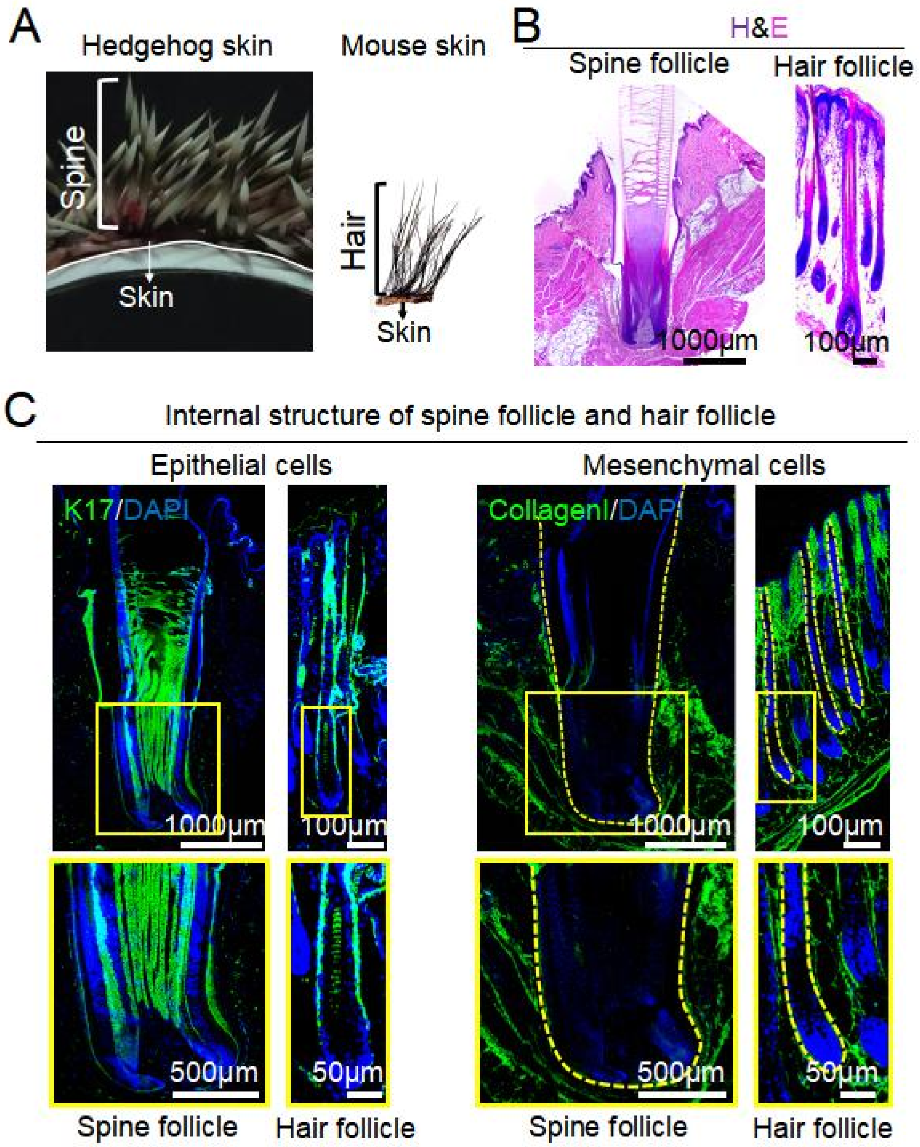

Figure S1. Morphology of spine follicles and hair follicles. A. Skin slices from hedgehog and mouse. B. HE staining shows the structure of spine follicles in hedgehogs and hair follicles in the mice. C. Immunostaining for K17 and CollagenI shows the epithelial and mesenchymal cells in the spine follicles and hair follicles.

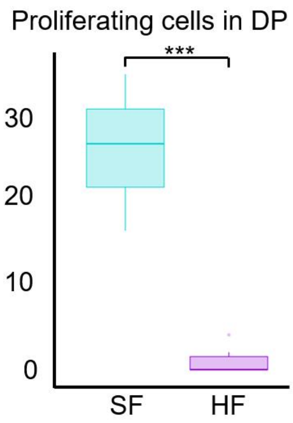

Figure S2. Statistical chart shows proliferating cells in the dermal papillar (DP). ***p < 0.001.

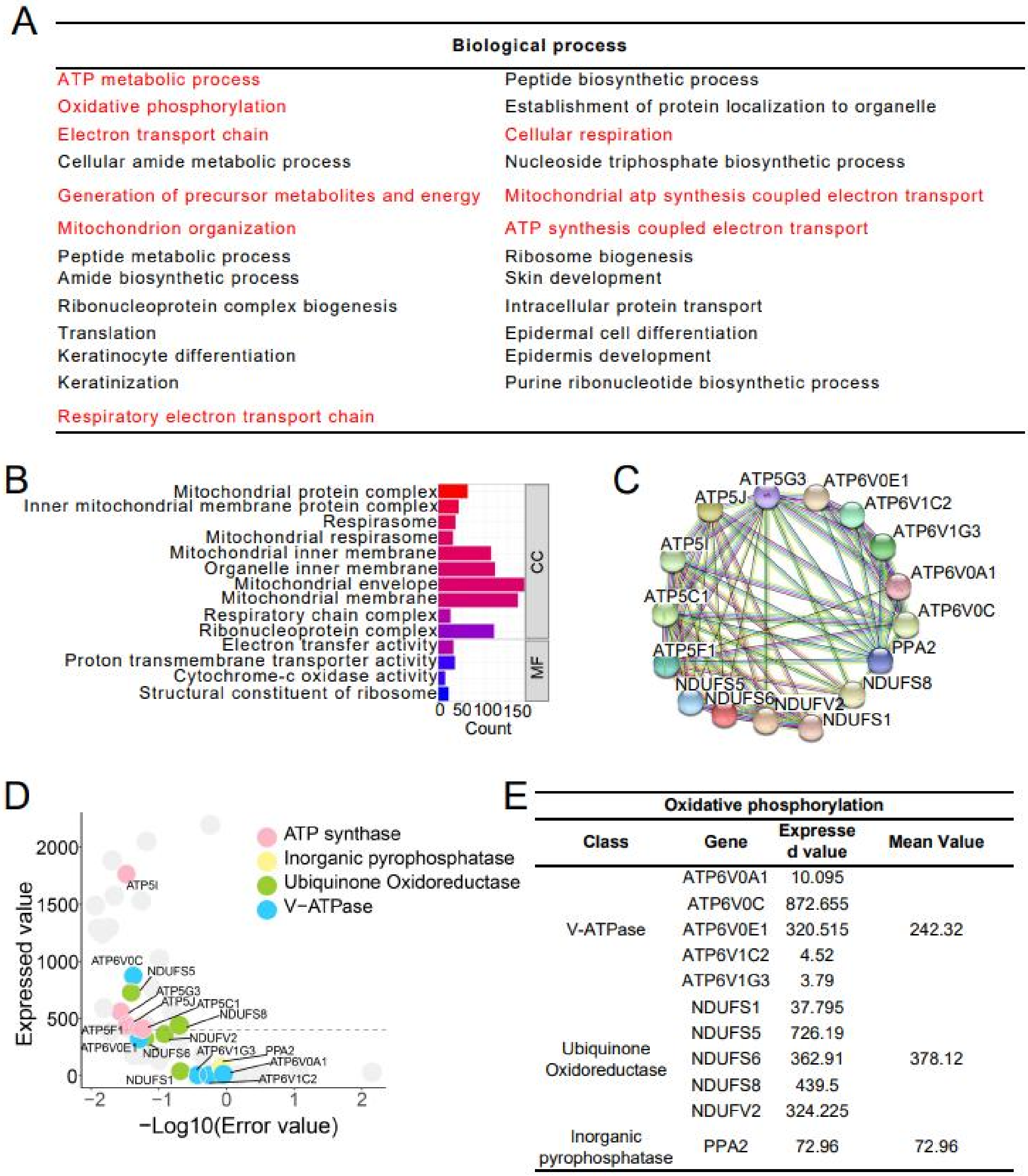

Figure S3. RNA-seq profiling and bioinformatic analysis reveal key molecules at the growth phase in the spine follicles. A. Biological processes GO analysis of genes only expressed in the spine follicles. B. Cell composition and molecular function GO analysis of genes only expressed in the spine follicles. C. Correlation between 16 related genes in oxidative phosphorylation. D-E. The expression value of genes encoding ATP synthase, Inorganic pyrophosphatase, ubiquinone oxidoreductase, and V-ATPase.

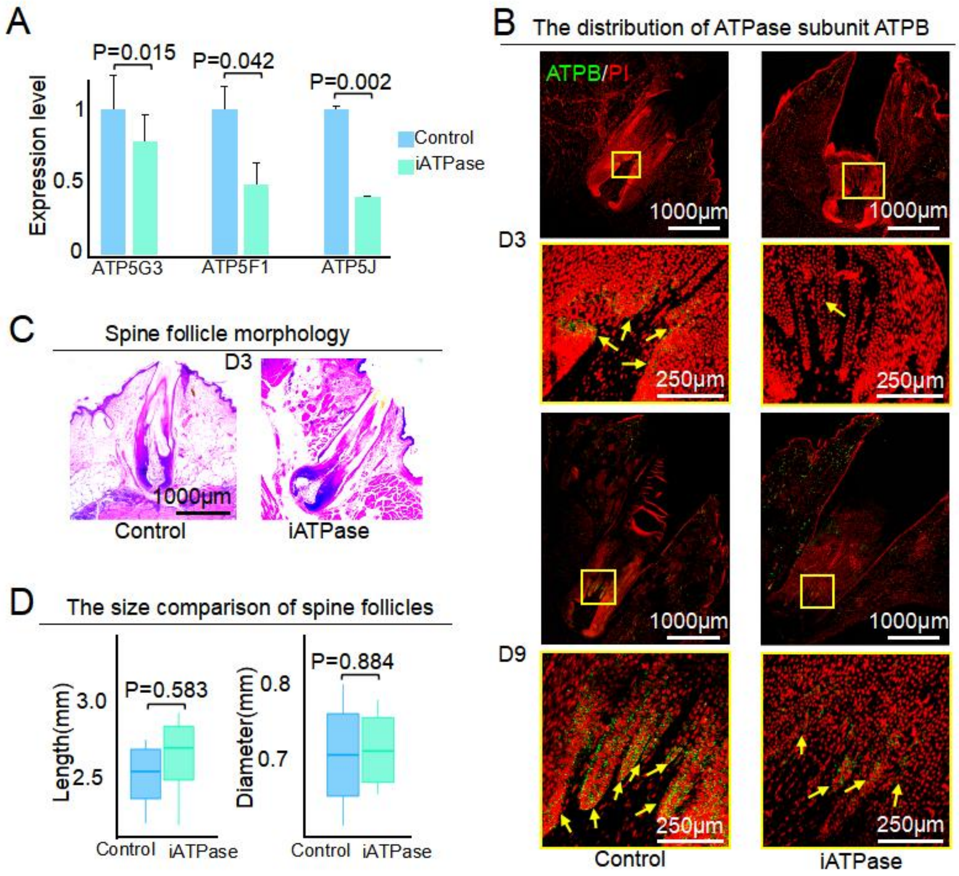

Figure S4. ATP synthase inhibitor treatment has no effect on spine follicle size on the third day. A. The expression level of ATPase related genes on D3 in the spine follicle. B. Immunofluorescence staining shows that the distribution of ATPase subunit ATPB. C. HE staining shows the internal structure. D. Statistical chart shows the length and diameter of the spine follicles in the control group and the experimental group.

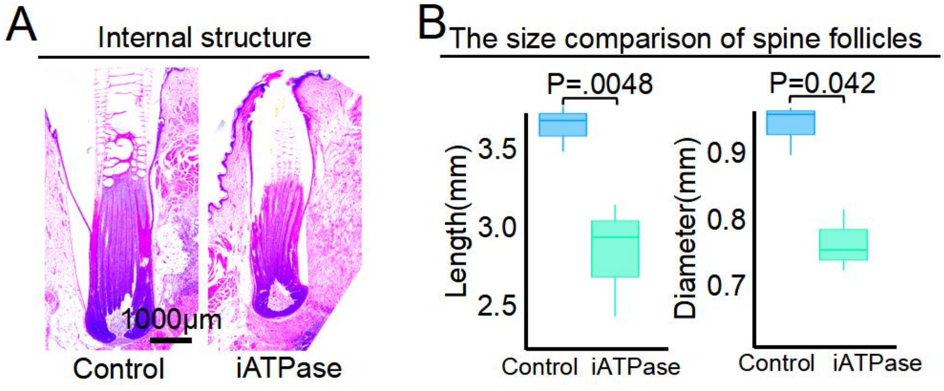

Figure S5. ATP synthase inhibitor treatment decreased spine follicle length and diameter on the ninth day. A. HE staining shows the structure of the spine follicle. B. Statistical chart shows the length and diameter of the spine follicles in the control group and the experimental group.

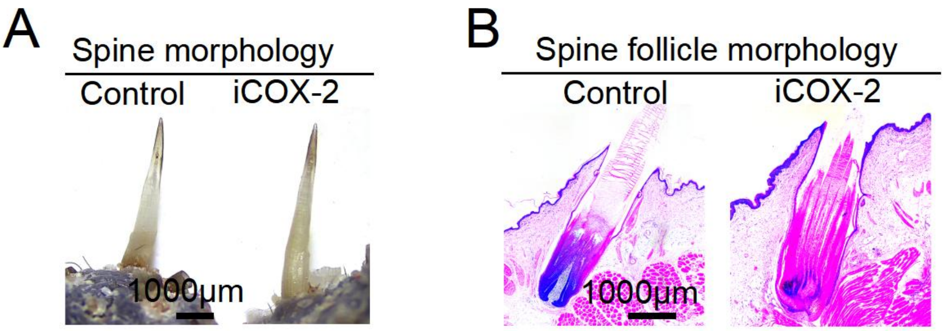

Figure S6. COX2 inhibitor treatment has no effect on spine follicle size on the third day. A. Spine morphology outside of the skin on the fifth day. B. HE staining shows the structure of the spine follicle on the third day.

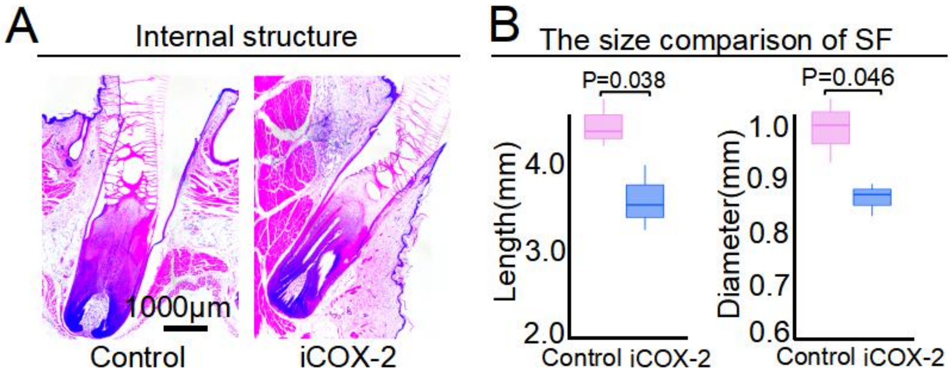

Figure S7. COX2 inhibitor treatment decreased spine follicle length and diameter on the ninth day. A. HE staining shows the structure of the spine follicle. B. Statistical chart shows the length and diameter of the spine follicles. SF: spine follicle.
